# Palm Olein Organogelation Using Mixtures of Soy Lecithin and Glyceryl Monostearate

**DOI:** 10.3390/gels8010030

**Published:** 2022-01-04

**Authors:** Sheah Yee Ghan, Lee Fong Siow, Chin Ping Tan, Kok Whye Cheong, Yin Yin Thoo

**Affiliations:** 1 School of Science, Monash University Malaysia, Jalan Lagoon Selatan, Bandar Sunway 47500, Malaysia; sheah.ghan@monash.edu (S.Y.G.); siow.lee.fong@monash.edu (L.F.S.); 2 Department of Food Technology, Faculty of Food Science and Technology, Universiti Putra Malaysia, Serdang 43400, Malaysia; tancp@upm.edu.my; 3 Department of Pharmaceutical Chemistry, School of Pharmacy, International Medical University, No. 126, Jalan Jalil Perkasa 19, Bukit Jalil, Kuala Lumpur 57000, Malaysia; CheongKokWhye@imu.edu.my; 4 Monash Industry Palm Oil Research and Education Platform, Monash University Malaysia, Bandar Sunway 47500, Malaysia

**Keywords:** palm olein, organogel, soy lecithin, glyceryl monostearate, mixture design

## Abstract

The present work investigated the interaction between soy lecithin (SL), glyceryl monostearate (GMS), and water in structuring palm olein (PO) to create an organogel having similar mechanical properties to commercial spread. Extreme vertices mixture design was used to optimize the composition of PO-based organogel. The resulting model showed a good fit to the predicted data with *R*^2^ ≥ 0.89. The optimum composition was 8% SL, 22% GMS, 28% water, and 42% PO (*w*/*w*) to produce a mean firmness of 1.91 N, spreadability of 15.28 N s^−1^, and oil binding capacity (OBC) of 83.83%. The OBC of optimized organogel was 10% higher than commercial spread product, and no significant difference was observed in the mechanical properties (*p* > 0.05). The microstructure, as well as the rheological and thermal properties of the optimized organogel were characterized. Fourier transform infrared analysis indicated that hydrogen bonding and van der Waals interactions were the key driving forces for organogelation. The mixture of SL and GMS favored the formation of β′ + β form crystals with a predominance of the β′ form. These results have important implications for the development of PO-based organogel as a potential fat replacer in the production of low-fat spread.

## 1. Introduction

Organogelation is a new way to structure liquid oil, by dissolving organogelators that could impart solid-like functional characteristics in an oil phase [1]. The formed organogels are thermodynamically stable, viscoelastic, and biocompatible [2]. They have a semi-solid structure that consists of three-dimensional networks formed by self-assembly of organogelators molecules to immobilize the continuous phase (edible oil). With edible oils as the main component, organogels have attracted interest from the food industry for replacing unhealthy partially hydrogenated fats. As such, the organogel fat replacer needs to possess similar characteristics to the partially hydrogenated fats.

A number of studies have been conducted on organogels toward food, cosmetic, and pharmaceutical applications in recent years [3]. The large number of identified organogelators can be categorized into polymeric and low molecular weight organogelators (LMWOs). Polymeric organogelators have limited applications in the food industry, and only ethyl cellulose has been intensively studied [4,5,6]. As for LMWOs, monoacylglycerols constitute a big market because they are cheap and commonly used as emulsifiers in foods [2,7]. Another important organogelator is lecithin, which is isolated and purified from soya beans or eggs. Lecithin has been extensively used as a viscosity modifier and stabilizer in sectors of the food industry. It is now also used in organogel preparation [8,9,10].

The influence of different organogelators on the physical properties of organogels has been addressed previously. However, most of those studies focused on the use of a single organogelator to structure the oil, while the use of organogelator mixtures could further improve the rheological and physical properties of food products. According to Lupi et al. [7], organogels produced using a mixture of glyceryl stearate and policosanol yielded higher dynamic moduli than pure organogelator gel. Lecithin has been shown to enhance the gelation of organogel by working with α-tocopherol [11], sitosterol [10], and sucrose esters [9]. In addition, 50% of the oil migration problems in chocolate products have been reduced by using a mixture of γ-oryzanol and β-sitosterol (overall, 25% of structurant) in organogel [12]. The mixed organogelator system has demonstrated the ability to alter the physical properties of organogel in a way that strongly depends on the ratio of the organogelator mixture [9,13]. Considering these observations, optimization of the organogel composition using a mixture of soy lecithin (SL) and glyceryl monostearate (GMS) as organogelators could achieve the desired physical properties.

Mechanical attributes such as firmness, spreadability, and oil separation are key physical properties determining the shelf life and consumer acceptance of food products [12]. Identified factors that significantly influence the mechanical properties of organogels include the type and polarity of the liquid oil, concentration of organogelators, and presence of water and co-surfactants [1,14,15,16]. However, most existing studies only analyzed the effect of each individual variable on the gel structure, while there are few coherent studies of the formulation-dependent mechanical properties of organogels. To improve the suitability of organogel as a fat replacer, the current work aims to control and modulate the mechanical properties of an organogel for future applications.

To eliminate trans fatty acids from the food system, there have been proposals to use palm oils and palm oil fractions to replace partially hydrogenated fats. Palm olein (PO) is fractionated from palm oil as a mixture of 45.3% saturated and 52.7% unsaturated fatty acids. It also contains various bioactive components including tocotrienols, tocopherols, and β-carotene [17]. Due to this typical balanced composition of fatty acids and the associated potential health benefits of bioactive components, PO remains widely used in the food industry despite the urges to replace saturated fatty acid. Nevertheless, to date there has been only limited development of PO-based organogels. New formulations of PO-based organogels using a mixture of SL and GMS could represent a major development in this regard.

Specifically, in order to formulate a PO-based organogel with mechanical properties comparable to commercial spread, the present study investigates the influence of specific components (namely the concentrations of SL and GMS, and the amounts of water and PO) on selected response variables of the organogel: the firmness, spreadability, and oil binding capacity (OBC). Firstly, the possible interactions between components in the mixed organogel were determined, and the composition was optimized using extreme vertices mixture design. This was followed by evaluation of the characteristics of optimized organogel, namely the microstructure, polymorphism, and structural and thermal properties. The optimized composition provides useful information for developing PO-based organogel as a fat replacer for a trans-fat-free spread product.

## 2. Results and Discussion

### 2.1. Model Fitting and Regression Modelling

Mixture design has been widely used in food production for composition optimization. This process is faster and more economical, and all the potential factors could be evaluated simultaneously and systematically [18,19,20,21]. In model fitting, the best model is usually fitted based on a high *R*^2^ (i.e., *R*^2^ > 0.70) and no significant difference (*p* > 0.05) in the lack-of-fit test [22]. Table 1 summarizes the experimental runs, their factor combinations, and the mean values of response variables. The best fitting model for each of the response variables was obtained using multiple regression with backward elimination. The accuracy of the models was estimated by *R*^2^, adjusted *R*^2^, predicted *R*^2^, and lack-of-fit, as shown in Table 2.

The *R*^2^ values for firmness, spreadability, and OBC models were observed to be 0.9412 (94%), 0.9699 (97%), and 0.8970 (90%), respectively. This indicates excellent fit of the generated polynomials to the experimental data, in which a high variability in the responding variables was attributed to the independent variables. These models showed no significant lack of fit (*p* > 0.05), suggesting that they fit the experimental data well. Contrarily, the adjusted *R*^2^ provides an adjustment to *R*^2^ based on the independent variables that are significant in the model, whereas the predicted *R*^2^ is used to evaluate how accurate the model is in predicting the response for future observations. All adjusted and predicted *R*^2^ values were found to be close to the actual *R*^2^, again showing the excellent fit of the models to the responses (Table 2). Therefore, the three different models generated for firmness, spreadability, and OBC are suitable for representing the experimental data. The quadratic polynomial equation for each response is as follows:

For firmness:

*Y*_1_ = 26.12*X*_1_ + 19.24*X*_2_ − 1.90*X*_3_ + 2.08*X*_4_ − 40.68*X*_1_*X*_3_ − 35.99*X*_1_*X*_4_ − 28.16*X*_2_*X*_4_

For spreadability:

*Y*_2_ = 200.50*X*_1_ + 216.20*X*_2_ − 5.30*X*_3_ + 26.20*X*_4_ − 290.30*X*_1_*X*_3_ − 270.30*X*_1_*X*_4_ − 366.10*X*_2_*X*_4_

For OBC:

*Y*_3_ = −326.80*X*_1_ + 206.10*X*_2_ + 66*X*_3_ + 20.50*X*_4_ + 634*X*_1_*X*_3_ + 736.50*X*_1_*X*_4_

### 2.2. Interaction between Organogel Components and Their Effect on Firmness, Spreadability, and OBC

The firmness of an organogel is defined as the maximum force required to deform its structure [23]. On the other hand, spreadability is used to measure how easy it is to apply a spread onto a surface. Spreadability decreases with the increasing firmness [24]. An organogel with low OBC releases the oil easily from the food matrix during chewing, which causes undesirable changes to the texture and sensory properties of the food [2]. Thus, the amount of oil released after centrifugation could be used to measure OBC as a quality indicator of organogel formation.

**Figure 1:**
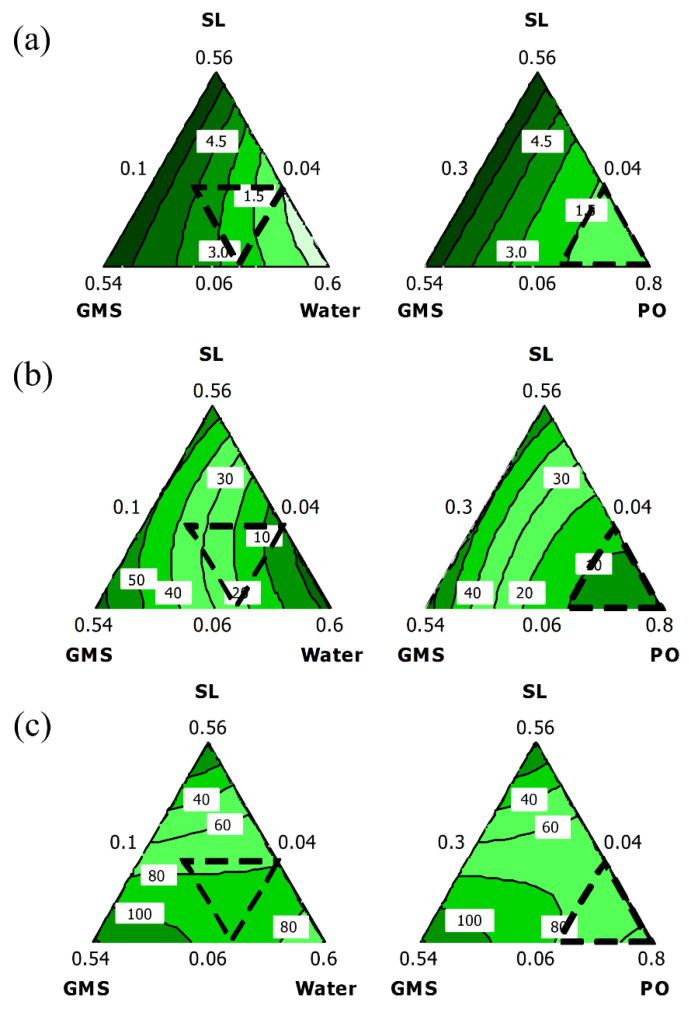
Mixture contour plots of predicted responses of the formulations: (**a**) firmness, (**b**) spreadability, and (**c**) oil binding capacity (OBC).

The interaction between different components in the organogel and their effects on the response variables could be explained from the mixture contour plots. Mixture contour plots for all three response variables (firmness, spreadability, and OBC) are shown in Figure 1a–c. Each vertex of the triangle represents a factor, while different points within the triangle indicate response data due to the combination effects of the mixture. The area of the contour plot beyond the dotted lines represents ‘out of constraint’ region, whereas the area within the dotted lines represents the region for actual experimental mixtures.

Among the mixture components, both SL (*X*_1_) and GMS (*X*_2_) had high positive linear coefficients in the suggested regression models of firmness and spreadability (Table 2). An increased concentration of SL or GMS improved the firmness and spreadability of the organogels (Figure 1a,b). These findings provide evidence that SL and GMS have excellent organogelation properties. They could self-aggregate to form a three-dimensional network, which further improved the structural integrity of the organogels [25,26]. On the contrary, as the amount of water (*X*_3_) increased, the firmness and spreadability decreased linearly. Increasing the amount of water caused instability in the organogel and eventually led to phase separation. A similar observation was reported by Angelico et al. [27]. This is very likely due to changes in the molecular arrangement, in which the water hinders the self-assembly of organogelators and eventually reduces the firmness of the organogel.

The interactions between SL and water, SL and PO, and GMS and PO decreased the firmness and spreadability of the organogels (Table 2 and Figure 1a,b). According to Li et al. [28], the gelling ability of an organogelator depends on its solubility in the solvent. When the organogelator is highly soluble in the solvent, a solution would form; while a highly insoluble organogelator will precipitate [29]. The hydrophile-lipophile balance (HLB) values of SL and GMS are 7 and 3.8, respectively [14,30]. SL, which has a high HLB value, shows a higher affinity toward hydrophilic compounds, allowing water molecules to bind to it and disrupting the arrangement of SL. Hence, when the amount of water was increased, the gelling capability of SL decreased, which further reduced the firmness and spreadability of the organogel. Conversely, GMS has a higher solubility in non-polar solvents. With a large volume of PO, GMS tends to dissolve in bulk oil, and its reduced ability to entrap the oil leads to the formation of a soft gel. In short, a balance between solubility and insolubility of organogelators in the solvent is a crucial factor in the organogel formation.

On the other hand, all the organogels prepared showed OBC values ranging from 50.03 to 91.53%. The OBC value increased with higher proportion of GMS in the formulation (Figure 1c). In a hydrophobic environment, GMS tends to form inverse lamellar phases by compressing its hydrophilic head group in the middle of the bilayer, with further interaction between its hydrophobic tails with the oil. This leads to the formation of a continuous network that increases the structural integrity of the organogel and reduces the oil mobility. The factors SL × GMS (*X*_1_*X*_2_), GMS × water (*X*_2_*X*_3_), and water × PO (*X*_3_*X*_4_) did not show significant effects (*p* > 0.05) in all regression models. This indicates that changes in these three combinations would not strongly impact the mechanical properties of the organogels.

Overall, the values of firmness, spreadability, and OBC increased with increased levels of SL (*X*_1_) and GMS (*X*_2_), along with a small amount of water (*X*_3_) and PO (*X*_4_) in the formulation (Figure 1). In other words, the organogelators (SL and GMS) play important roles in modifying the mechanical properties of an organogel, owing to the interaction between the organogelators and the solvent phase (water and PO). These results show that the optimum composition varies for different mechanical properties, and therefore a response optimizer is essential to obtain a compromised optimum formulation.

### 2.3. Optimization and Verification of Results

The criterion for choosing a suitable mixture combination was based on the optimal firmness value (1.81–2.16 N), moderate spreadability (14.7–17.64 N s^−1^), and maximal OBC. These ranges were obtained for commercial spreads from different manufacturers. Based on these values, optimization of the organogel composition was performed using the multi-response optimizer function in Minitab software. In order to obtain the best balance among the different responses, a composite desirability (D) with values in the range of 0 (unacceptable response value) to 1 (desirable value) was used to optimize various responses simultaneously [2]. The best optimum values for SL, GMS, water, and PO in the organogel were found to be 8, 22, 28, and 42%, respectively (Table 3). A composite desirability of 0.9223 was obtained, indicating that this composition achieved favorable results for all responses. To verify the established mathematical models, *t*-test was conducted to compare the experimental and predicted values for each response (Table 3). The presented models demonstrated high accuracy and validity in predicting the response variables, as the difference between the two values is not significant.

Comparison was also made between the optimized organogel (SL/GMS/PO) and a commercial spread (firmness of 2.03 ± 0.06 N, spreadability of 15.81 ± 0.52 N s^−1^, and OBC of 76.26% ± 0.92%). No significant difference was observed in the firmness and spreadability values (*p* > 0.05) between SL/GMS/PO and the commercial spread. Meanwhile, the OBC of the former was 10% higher, indicating a better ability to entrap the oil and reduce the occurrence of syneresis (separation of oil). According to Codex Alimentarius, the fat content of a spread product must be less than 80% (WHO/FAO 2007). SL/GMS/PO, which contains 42% oil, meets the requirement for a low-fat spread. Therefore, further characterization of SL/GMS/PO was conducted to determine its physical properties prior to food application.

### 2.4. Evaluation of Prepared Organogels

#### 2.4.1. Polarized Microscopy Analysis

**Figure 2:**
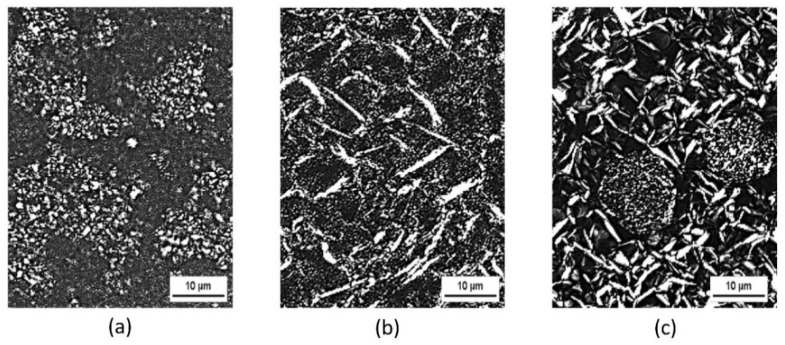
Polarized micrograph of (**a**) SL/PO, (**b**) GMS/PO, and (**c**) SL/GMS/PO organogel under high magnification (40×).

The polarized micrographs of SL/PO, GMS/PO, and SL/GMS/PO organogels are depicted in Figure 2. During organogel preparation, SL, which contains phospholipid molecules, self-assembled into spherical reverse micelles that grew into giant cylindrical micelles. These micelles tend to overlap, interpenetrate, and entangle among each other to form a three-dimensional network (Figure 2a). GMS appears as rod-like aggregates in organogels, as shown in Figure 2b. When a mixture of GMS and SL was used in organogelation, water bound to the hydrophilic phosphate head of the SL molecules and acted as a bridge between two adjacent SL. GMS molecules were connected to each other and intermeshed around the entangled reverse micelles of SL, developing a structural gel network that physically entrapped the liquid oil (Figure 2c). This is similar to the result from Raut et al. [31], where SL molecules arrange themselves at the interface between oil and water compounds to reduce the interfacial tension, forming a relatively stable emulsion of SL/GMS/PO organogel.

#### 2.4.2. FTIR

**Figure 3:**
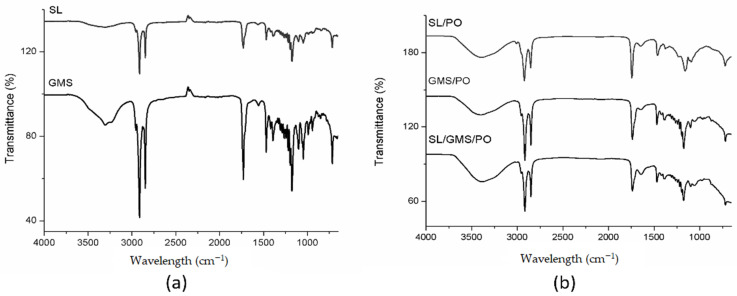
FTIR spectra of (**a**) pure organogelators and (**b**) SL/PO, GMS/PO, and SL/GMS/PO organogels.

FTIR analysis was conducted at room temperature (25 °C) to determine the intermolecular interactions amongst individual components within the organogel sample. Based on Figure 3a, both SL and GMS gelators show a medium, broad peak at around 3390 cm^−1^, which represents the stretching vibration of -OH group. Whereas, the absorption peaks at 2848 and 2914 cm^−1^ represent the CH asymmetric stretch in CH_3_ and CH_2_, and the CH symmetric stretch in CH_2_ and CH_3_, respectively. A similar peak (3390 cm^−1^) was also observed in SL/PO, GMS/PO, and SL/GMS/PO organogels.

Figure 3b, which likely indicate presence of hydrogen bonding between the molecules. However, the absorption bands of symmetric and anti-symmetric CH_2_ were shifted slightly from 2848 and 2914 cm^−1^ to 2850 and 2916 cm^−1^ for organogels. These small peak shifts between pure organogelators and organogels were reported to be due to the presence of van der Waals interactions, which decrease the fluidity of the alkyl chains [32,33]. Although both hydrogen bonding and van der Waals interactions are present according to the FTIR data, the former plays a more important role in organogel formation due to the presence of water. The SL/GMS/PO organogel showed a similar spectrum to the SL/PO and GMS/PO organogels. There was no peak shift, peak disappearance, or new peak formation in the rest of the spectra. This clearly indicates that the combination of SL and GMS in the organogel does not alter the chemical functionality of each component, and that the organogelation process involves only physical interactions.

#### 2.4.3. XRD

**Figure 4:**
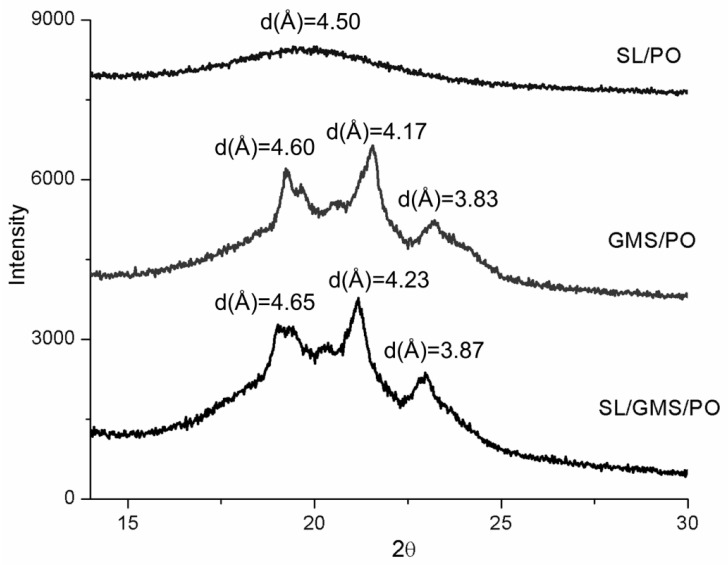
The XRD graphs of SL/PO, GMS/PO, and SL/GMS/PO organogels.

The XRD patterns of the organogels at ambient temperature are shown in Figure 4. A hump was observed in SL/PO, indicating micellar aggregation in the system. This result is consistent with the findings reported by Brad and Chen [34], where SL-organogels showed an amorphous nature. GMS/PO shows multiple diffraction peaks at d = 3.8 − 4.2 and 4.6 Å, indicating a mixture of β′ and β polymorphs. The presence of GMS also induced the formation of polymorphic β′ + β crystals in SL/GMS/PO. A higher peak intensity at 4.6 Å was observed in GMS/PO added with 30% GMS when compared to SL/GMS/PO added with 22% GMS. This suggested that the addition of GMS into PO favored the formation of β′ + β form crystals, with a predominance of the β’ form (as indicated by the strong peaks at 3.8 and 4.2 Å). This β′ form is desirable because it provides a homogeneous, smooth, creamy, and fine texture in spread products [35].

#### 2.4.4. Rheology

**Figure 5:**
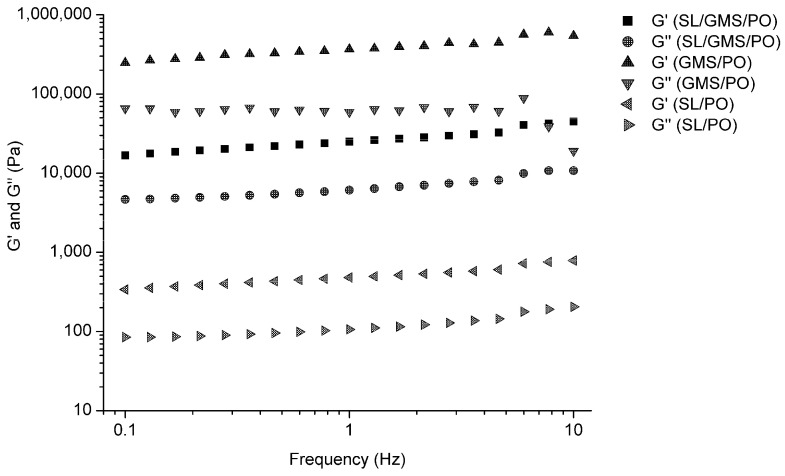
Rheological-frequency sweep test of SL/PO, GMS/PO and SL/GMS/PO organogels.

Rheological analysis was performed to determine the viscoelasticity of SL/PO, GMS/PO, and SL/GMS/PO. This was done by evaluating the changes in the storage modulus (G′) and loss modulus (G″). G′ reflects the solid-like component in the rheological behavior, whereas G″ represents the viscosity of the material [36]. In Figure 5, the results of frequency sweep test showed that all the samples had higher G′ than G″ throughout the measurement. Hence, they showed gel-like behavior at room temperature (25 °C), which is similar to the rheological properties of semisolid spread products such as yoghurt and cream cheese [36]. The lower dynamic moduli of SL/GMS/PO indicate that this gel is easier to spread as compared to GMS/PO. Nevertheless, SL/PO is too soft to be applied as a spread product. The absence of a cross-over point between G′ and G″ at high frequency confirms the absence of gel-sol transformation, when force is applied on the organogels at room temperature. This is likely due to the formation of a strong gel network that entangles the oil in a firm gel [37].

**Figure 6:**
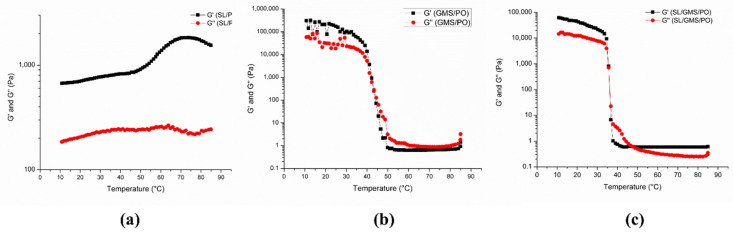
Rheological-temperature ramp test of (**a**) SL/PO, (**b**) GMS/PO, and (**c**) SL/GMS/PO organogels.

The thermal behavior of all the samples was studied by cooling the samples from 80 to 10 °C (Figure 6). At temperatures above 50 °C, the G′ value of both SL/PO and SL/GMS/PO was higher than G′′, showing the ability of organogels to maintain their elastic properties when exposed to high temperature. This is owing to the interaction between SL and water in both organogels, in which the water is bound strongly with SL in the hydrophobic environment. Moreover, the addition of water to lecithin organogel induces the formation of coexisting gel and lamellar phases [27]. This explains the elastic properties shown by SL/PO and SL/GMS/PO. On the contrary, the GMS/PO organogel showed a viscous behavior, and it melted completely at high temperature.

**Figure 7:**
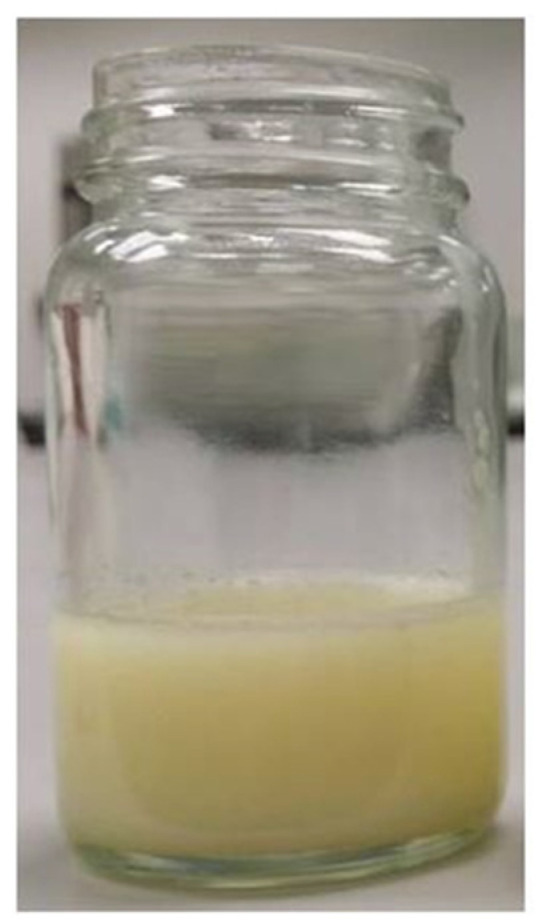
Organogel at room temperature.

At temperatures from 45 to 55 °C, a sudden increase in the complex modulus curve was observed in GMS/PO and SL/GMS/PO organogels, indicating the beginning of organogel crystallization. During this cooling process, the gel network structure began to form due to the increased interaction between the organogelator molecules and the aggregation of the molecules over the entire sample. The temperature at which a crossover is observed between G′ and G″ is known as the gelation point (Tg), where the system begins to transform from a free-flowing liquid phase to a gelled phase [32]. The measured Tg values of GMS/PO and SL/GMS/PO were 43 and 38 °C, respectively. This observation suggests that the presence of SL molecules in SL/GMS/PO may interfere with the self-assembly of GMS during organogelation, which causes the onset of crystallization in SL/GMS/PO later. At temperatures below 30 °C, G′ increases slightly due to the reduction in molecular mobility at a lower temperature. Hence, both GMS/PO and SL/GMS/PO behave as solid-like gel materials at room temperature (25 °C) (Figure 7).

#### 2.4.5. Differential Scanning Calorimetry (DSC) Analysis

**Figure 8:**
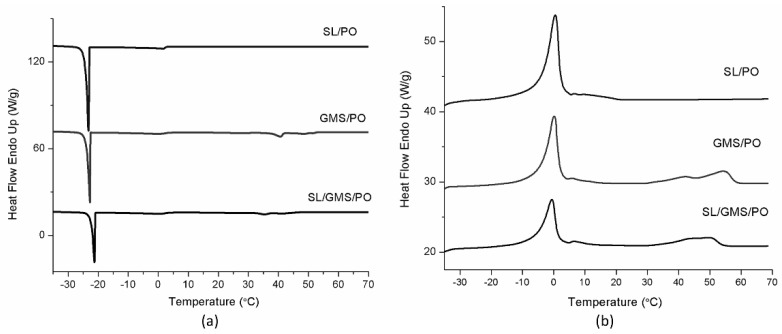
(**a**) DSC crystallization thermogram and (**b**) DSC melting thermogram of SL/PO, GMS/PO and SL/GMS/PO organogels.

Figure 8 shows the DSC thermograms of all the prepared organogels. Based on Figure 8a, the onset of crystallization for GMS/PO and SL/GMS/PO occurred at 52.67 and 45.32 °C, respectively. These temperatures were similar to the temperature at which a sudden increase in the complex modulus was found in the rheological analysis (Figure 6b,c). Hence, it is strongly believed that the first transition peak in the thermogram was due to the self-assembly of organogelator, which acted as a precursor for crystal growth and aggregation [32].

Nevertheless, the crystallization and melting peaks of SL are not notable in the SL/PO thermogram. A similar observation was reported by Brad and Chen [34] and Yusuf et al. [38], who attributed the loss of these peaks to the presence of bound water in SL, which affected the crystalline nature of SL. This bound water could only be released from the SL by heating the sample to 251 °C. As for SL/GMS/PO, its DSC thermogram exhibited combined characteristics of SL and GMS. Based on Figure 8b, the melting thermogram of SL/GMS/PO exhibited endothermic peaks at −0.56 °C (melting of water) and 49.62 °C. The peak at 49.62 °C indicated the transition when the gel phase was converted to a liquid crystalline state. Again, this result is consistent with the rheological results, where the structural integrity of SL/GMS/PO was compromised at 50 °C, which caused a reduction in the elasticity.

## 3. Conclusions

In this study, extreme vertices mixture design was successfully utilized to optimize the composition of PO-based organogels using mixed organogelators to achieve desired mechanical properties for spreads. The optimum proportions obtained was 8% SL, 22% GMS, 28% water, and 42% PO (*w*/*w*), which produced an organogel with high OBC, strong gel network, and mechanical properties comparable to the commercial spread. This organogel (SL/GMS/PO) showed structural and rheological properties intermediate between those of SL/PO and GMS/PO. However, its crystal polymorph and thermal properties seem to be closer to GMS/PO. The physical properties of SL/GMS/PO were strongly affected by the proportions of SL and GMS used. These results contribute to the development of PO-based organogels formed using mixed organogelators. Further study is being carried out to improve the storage stability of SL/GMS/PO.

## 4. Materials and Methods

### 4.1. Materials

Palm olein (PO, iodine value (IV) = 56) was acquired from MOI Foods Malaysia Sdn Bhd (Klang, Malaysia). Soy lecithin (SL) was purchased from R&M Chemicals (R&M Marketing, Essex, UK), and glyceryl monostearate (GMS) was acquired from a local food ingredient supplier (Oleofine Organics Sdn. Bhd., Shah Alam, Malaysia). A commercial spread product of reduced fat (Naturel, Lam Soon, Shah Alam, Malaysia) was purchased from Tesco supermarket (Puchong, Malaysia). This commercial spread product contains palm oil with a total fat content of 55.5%.

### 4.2. Experimental Design

Mixture design is commonly used to study the relationship between mixture components to determine the optimum composition using desirability approach [18,19,21]. In the present study, the proportional level of four selected factors: SL (*X*_1_), GMS (*X*_2_), water (*X*_3_), and PO (*X*_4_) were determined and analyzed using extreme vertices mixture design performed with Minitab 17 (Minitab LLC, State College, PA, USA). The lower and upper weight limits for the SL, GMS, water, and PO contents were 6–30%, 4–30%, 10–40%, and 30–80%, respectively, summing to 100% in each final formulation. All the experimental runs, factor levels, and responding variables are presented in Table 1. Multiple regression with backward elimination was conducted to determine the best-fitting models. The polynomial equation for quadratic regression model was as follows:

(1) $$Y=\beta_{1}X_{1}+\beta_{2}X_{2}+\beta_{3}X_{3}+\beta_{1}\beta_{2}X_{1}X_{2}+\beta_{1}\beta_{3}X_{1}X_{3}+\beta_{2}\beta_{3}X_{2}X_{3}$$

where *Y* is the predicted response (firmness, spreadability, and OBC), β is the regression coefficients for each linear and quadratic effect terms, and *X* is the factor considered (SL, GMS, water, and PO). The analysis of variance (ANOVA) was used to determine the quality of the response models. The statistical coefficients used to interpret the models were the coefficient of determination (*R*^2^), the adjusted *R*^2^, and the predicted *R*^2^. Besides that, the lack-of-fit test was applied to indicate the probability of the model’s failure to predict the experimental data. The value obtained should not be significant, where *p* > 0.05.

### 4.3. Preparation of Organogels

A total of 29 organogel formulations were prepared according to the extreme vertices mixture design (Table 1). Each formulation was prepared by dissolving SL and GMS in PO at 65 °C in a water bath. Water was added part by part, followed by mixing for 10 min to form a homogeneous solution. The sample was then stored overnight at 25 °C to ensure complete gelation. The tubes were inverted to ensure the formation of organogel [8].

### 4.4. Mechanical Properties

The mechanical strength of organogels was evaluated in terms of firmness and spreadability. Both analyses were performed using the TA-XT plus texture analyzer (Stable Microsystems Ltd., Godalming, Surrey, UK) at room temperature (25 °C). Twenty grams of the organogel sample was first filled in a glass bottle (5.8 cm × 45 mm diameter). The firmness of the sample was then determined with a cylindrical probe (P/0.5R with 1/2 in diameter). The measurements were done in the compression mode, in which the probe penetrated 12 mm into the sample (2 mm/s), and returned to the initial position at the same speed.

For the spreadability test, a male 90° cone probe and female perspex cone (sample holder) were used [39]. Prior to the test, organogel samples were filled into the female cone and fixed on a heavy-duty platform of the instrument. Measurement was done by compressing the sample using the male cone probe, which shifted by a distance of 23 mm at the test speed of 3 mm/s. The probe then withdrew at 10 mm/s back to the original position.

### 4.5. Oil Binding Capacity (OBC)

The ability of the organogel to entrap oil was determined following the method described by Martins et al. [40]. Five grams of the melted organogel was added to a centrifuge tube and cooled at 25 °C for 1 h. Subsequently, the sample was centrifuged at 9000 rpm (25 °C) for 20 min, and the supernatant was removed. The tube was weighed both before centrifugation and after the removal of excess oils. The OBC was calculated using the following formulas:

(2) $$\%\;\mathrm{Released}\;\mathrm{oil}=\frac{\mathrm{Mass}\;\mathrm{of}\;\mathrm{the}\;\mathrm{oil}\;\mathrm{released}}{\mathrm{Mass}\;\mathrm{of}\;\mathrm{the}\;\mathrm{sample}\;\mathrm{before}\;\mathrm{centrifugation}}\;\times \;100\%$$

% OBC = 100% of released oil

### 4.6. Optimization and Verification of Models

Multiresponse optimization was used to determine the optimum formulation of organogel based on composite desirability [2]. Model verification was performed by conducting confirmative test on the organogel prepared with the optimum composition. *t*-test was further conducted to compare the experimentally determined values with those predicted by the multiresponse optimizer.

### 4.7. Organogel Characterization

The molecular, rheological, and thermal properties of the organogels (both the optimized and those using a pure organogelator) were studied. The organogels prepared using pure SL, pure GMS and the optimized organogel are coded ‘SL/PO’, ‘GMS/PO’, and ‘SL/GMS/PO’ samples, respectively. These organogels are prepared via fluid-filled fiber mechanism with 28% of water in SL/PO and GMS/PO.

#### 4.7.1. Polarized Microscope Observation

Microstructure images of the organogels were obtained under a polarized light microscope (Olympus, model BX51, Tokyo, Japan) equipped with a digital camera (Nikon, DS-Filc, Tokyo, Japan). Initially, the organogels were melted at 65 °C. One drop of the melted organogel was then deposited on a glass slide and covered with a coverslip. Prior to microscope observation, the slides were stored overnight at 25 °C to ensure proper crystallization. The organogels were observed at 40× magnification at room temperature (25 °C), and the images were obtained using NIS-Element Imaging Software (Version 4.20, Nikon, Tokyo, Japan).

#### 4.7.2. Fourier Transform Infrared (FTIR) Spectroscopy

Organogel samples were placed directly on the attenuated total reflection (ATR) crystal. FTIR spectra of the organogels were measured at 4 cm^−1^ resolution from 650 to 4000 cm^−1^ using a Varian 640-IR FTIR spectrophotometer (Varian Medical System, Palo Alto, CA, USA). Each spectrum was composed of 32 scans at room temperature (25 °C) [8].

#### 4.7.3. X-ray Diffraction (XRD) Analysis

The polymorphic forms of the fat crystals were determined at 25 °C with a D8 Discover X-ray diffractometer (Bruker, Karlsruhe, Germany) fitted with Cu-Kα radiation (*k* = 1.5418 Å) at the voltage and current of 40 kV and 40 mA, respectively. The samples were analyzed at 2*θ* angles of 5° to 50° with a scanning rate of 2° 2*θ*/min. The X-ray peak and its width at half maximum (FWHM) were calculated using Diffrac. EVA (Bruker) software [41].

#### 4.7.4. Rheological Analysis

Rheological properties of the organogels were determined using a rheometer (Anton Paar MCR302, Saint Laurent, QC, Canada) equipped with a temperature control unit. Using a parallel plate geometry (PP 25/s), the strain sweep tests were performed at a constant frequency of 1 Hz and at strain amplitude varying from 0.1 to 100 Pa to investigate the linear viscoelastic region (LVR) of organogels. Subsequently, frequency sweep test was performed at a fixed strain (10 Pa) with frequency 0.1–10 Hz. Both strain sweep and frequency sweep tests were carried out at room temperature (25 °C). The temperature ramp test was performed at 1 Hz frequency and the temperature range from 10 to 80 °C with cooling rate 1 °C/min, to study the thermal sensitivity of organogels. The G′ and G′′ values (storage modulus and change modulus, respectively) for each sample were obtained.

#### 4.7.5. Differential Scanning Calorimetry (DSC)

The crystallization and melting thermograms were obtained by using a differential scanning calorimeter (DSC; Pyris 4000 DSC; Perkin Elmer, Waltham, MA, USA) equipped with a refrigerated cooling unit. About 10–12 mg of sample was loaded in a sealed pan, and an empty aluminum pan was used as reference. Experiments were conducted using a nitrogen flow rate of 20 mL/min. The samples were first heated at 80 °C for 10 min to remove all the fat crystal memory, followed by cooling (5 °C/min) to −50 °C and re-heating to 80 °C at the same rate [3].

### 4.8. Statistical Analysis

Each organogel sample was prepared in duplicate, and each sample analysis was repeated at least twice. The results were represented as mean ± standard deviation (SD). Data were analyzed statistically by one-way ANOVA, and Tukey’s test using the Minitab 17 software. The significant differences at a *p* < 0.05 level were used.

## Figures and Tables

**Table 1:** Experimentally measured response variables for organogels formulated with different proportions of soy lecithin (SL), glyceryl monostearate (GMS), water, and palm olein (PO).

Sample Number	Independent Variables (% *w*/*w*)				Response Variables		
	*X*_1_: SL	*X*_2_: GMS	*X*_3_: Water	*X*_4_: PO	*Y*_1_: Firmness (N)	*Y*_2_: Spreadability (N S^−1^)	*Y*_3_: OBC (%)
1	13.00	10.00	10.00	67.00	1.17	7.84	74.69
2	19.00	7.00	18.00	56.00	0.78	9.05	80.26
3	26.00	4.00	40.00	30.00	0.28	4.56	82.14
4	13.00	10.00	40.00	37.00	0.45	5.05	78.26
5	6.00	14.00	40.00	40.00	0.82	7.69	80.93
6	16.00	4.00	10.00	70.00	0.84	8.88	73.85
7	16.00	14.00	25.00	45.00	1.52	15.59	82.77
8	6.00	4.00	10.00	80.00	0.20	2.00	50.03
9	6.00	24.00	25.00	45.00	3.62	17.08	86.09
10	6.00	14.00	25.00	55.00	1.06	6.81	75.15
11	13.00	10.00	25.00	52.00	0.78	7.01	71.74
12	16.00	14.00	40.00	30.00	1.20	12.81	85.38
13	9.00	7.00	18.00	66.00	0.56	5.84	62.30
14	9.00	17.00	33.00	41.00	1.20	10.41	78.70
15	6.00	4.00	25.00	65.00	0.33	4.07	58.07
16	26.00	4.00	10.00	60.00	1.36	13.52	71.12
17	13.00	10.00	25.00	52.00	0.74	6.22	70.89
18	6.00	14.00	10.00	70.00	0.99	6.33	63.37
19	16.00	4.00	40.00	40.00	1.00	8.86	81.11
20	16.00	14.00	10.00	60.00	2.31	19.97	76.76
21	16.00	4.00	25.00	55.00	0.28	3.74	59.21
22	9.00	17.00	18.00	56.00	1.31	8.22	66.20
23	13.00	10.00	25.00	52.00	0.68	3.98	74.45
24	6.00	24.00	10.00	60.00	1.67	10.11	69.05
25	6.00	4.00	40.00	50.00	0.23	1.29	63.67
26	6.00	24.00	40.00	30.00	2.52	23.93	91.50
27	9.00	7.00	33.00	51.00	0.24	1.65	65.85
28	26.00	4.00	25.00	45.00	0.35	5.82	74.46
29	19.00	7.00	33.00	41.00	0.39	5.16	77.87

**Table 2:** Regression coefficients, *R*^2^, adjusted *R*^2^, and probabilities for the firmness, spreadability, and oil binding capacity (OBC) of organogels.

Independent Variables	Regression Coefficients		
	Firmness	Spreadability	OBC
Linear			
SL	26.12	200.50	−326.80
GM	19.24	216.20	206.10
Water	−1.90	−5.30	66.00
PO	2.08	26.20	20.50
Quadratic			
SL × Water	−40.68 *	−290.30 *	634.00 *
SL × PO	−35.99 *	−270.30 *	736.50 *
GMS × PO	−28.16 *	−366.10 *	-
*R* ^2^	0.9412	0.9699	0.8970
Predicted *R*^2^	0.8150	0.9231	0.8197
Adjusted *R*^2^	0.9216	0.9548	0.8712
Lack of fit			
*F* value	10.1400	0.3700	3.5300
*p* value	0.0930	0.9010	0.2430

* *p* < 0.05 indicates statistical significance.

**Table 3:** Optimum formulation composition, comparison of experimental results with predicted response.

Factors				Dependent Variables (Response)	Optimum Value		
SL	GMS	Water	PO		Experimental ^a^	Predicted	*p*-Value
8.00	22.00	28.00	42.00	Firmness	1.91 ± 0.04	1.91	0.923
				Spreadability	15.28 ± 0.66	15.68	0.372
				OBC	83.83 ± 4.14	85.15	0.568

^a^ Mean ± standard deviation (SD) of four determinations (*n* = 4).

## Data Availability

The data will be available from the corresponding authors on request.
